# Simulated Inherent Optical Properties of Aquatic Particles using The Equivalent Algal Populations (EAP) model

**DOI:** 10.1038/s41597-023-02310-z

**Published:** 2023-06-24

**Authors:** Lisl Robertson Lain, Jeremy Kravitz, Mark Matthews, Stewart Bernard

**Affiliations:** 1 Council for Scientific and Industrial Research, Cape Town, South Africa; 2 Bay Area Environmental Research Institute, Moffett Field, CA USA; 3 NASA Ames Research Center, Mountain View, CA USA; 4 Cyanolakes Pty. Ltd, Cape Town, South Africa; 5 South African National Space Agency, Cape Town, South Africa

**Keywords:** Ocean sciences, Applied optics

## Abstract

Paired measurements of phytoplankton absorption and backscatter, the inherent optical properties central to the interpretation of ocean colour remote sensing data, are notoriously rare. We present a dataset of Chlorophyll *a* (Chl *a*) -specific phytoplankton absorption, scatter and backscatter for 17 different phytoplankton groups, derived from first principles using measured *in vivo* pigment absorption and a well-validated semi-analytical coated sphere model which simulates the full suite of biophysically consistent phytoplankton optical properties. The optical properties of each simulated phytoplankton cell are integrated over an entire size distribution and are provided at high spectral resolution. The model code is additionally included to enable user access to the complete set of wavelength-dependent, angularly resolved volume scattering functions. This optically coherent dataset of hyperspectral optical properties for a set of globally significant phytoplankton groups has potential for use in algorithm development towards the optimal exploitation of the new age of hyperspectral satellite radiometry.

## Background & Summary

The aim of ocean optics in the context of understanding ocean productivity and climate change is to identify that portion of the water-leaving radiometric signal that is attributable to phytoplankton. The use of ocean colour data (whether from satellite, airborne sensors or in-water radiometry) to infer biogeochemical information from the in-water constituents requires detailed understanding not only of the absorption coefficient, which is readily measured *in situ*, but also of the sources of backscatter and its associated variability.

Community understanding of ocean optics has advanced exponentially since the early days of satellite oceanography, but the legacy of oversimplified marine particulate optical models persists in many of the approaches to algorithms for geophysical product retrievals. It is now well established that historical approaches to modelling marine particulate backscatter (e.g. homogenous sphere models such as Mie solutions) are no longer considered appropriate^1–4^ – and in fact, may have misled community understanding in terms of the sources of backscatter variability and particle composition in the ocean^3^.

The much-anticipated launch of NASA’s Plankton Aerosol Cloud ocean Ecosystem sensor (PACE) in 2024 signifies the dawn of a new age of hyperspectral radiometry requiring new hyperspectral algorithms to advance capability in retrieving aquatic optical properties. While there is no replacement for the scientific value of *in situ* datasets, they do not currently support this goal: many ocean regions are drastically undersampled, and while absorption is relatively well characterised there is a dearth of backscatter measurements, and only then at a few wavelengths. Synthetic data can be used – with the appropriate care - to complement and support the optimal exploitation of *in situ* measurements, and towards the development of powerful machine/deep learning and AI techniques for the retrieval of biogeochemical water parameters in challenging optical conditions.

Here we present a database of fully spectrally resolved algal inherent optical properties for use in constituent retrieval algorithm development and for input into radiative transfer models for water-leaving signal analysis and top of atmosphere sensitivity studies. It is critical that synthetic datasets are treated with care when intending to represent the natural environment, and to note that the strength of the Equivalent Algal Populations (EAP) model presented here is to demonstrate signal causality and investigate sources of optical variability, rather than to exactly represent *in situ* phytoplankton optics.

The EAP model is unique in two ways:

- It acknowledges that the absorption and backscatter coefficients of the in-water constituents contributing to the water-leaving signal are not independent of each other in nature, and are in fact causally related. This is a fundamental characteristic of aquatic particles often overlooked by models that handle absorption and backscatter separately.
- This biophysical consistency allows investigation of the relationship between phytoplankton intracellular Chl *a* density (*c*_*i*_) and phytoplankton intracellular carbon density (*C*_*i*_), i.e. Chl *a* to carbon ratios, as carried through into the optical properties.

The model has previously been used to investigate phytoplankton size retrievals via the inversion of water leaving radiometry^5^, demonstrate the impact of a gas vacuole on the IOPs of *Microcystis aeruginosa*^6^, generate a high biomass switching algorithm for MERIS^7^, as well as to create a global dataset of particulate size and phytoplankton carbon retrievals^8^. The sensitivity and effectiveness of different machine learning approaches to in-water constituent retrieval has been tested using EAP phytoplankton IOPs^9^, as well as the sensitivity of hyperspectral optical signals resulting from phytoplankton assemblage changes in the Southern Benguela upwelling region and in the Southern Ocean^10^. There is also a more technical investigation on the uncertainty introduced into radiative transfer simulations by approximating phytoplankton phase functions^2^, all facilitated by EAP spectral phytoplankton IOPs in conjunction with Hydrolight radiative transfer model (Sequoia, Inc).

By making some general EAP IOP outputs available to the community we hope to facilitate a new generation of hyperspectral biogeochemical algorithms for use with satellite radiometry, exploiting the respective advantages of both absorption and backscattering signals for the retrieval of phytoplankton optical properties while maintaining natural biophysical consistency. This approach holds notable potential for particulate carbon retrievals from radiometry, with recent work revealing that combined *a* and *b*_*b*_ approaches may improve carbon biomass estimates^11–13^ towards addressing the critical question of the trajectory of the global carbon pump^14,15^.

## Methods

### Model description and features

The comprehensive model description and derivation^4^, and a more user-friendly summary^10^, emphasises that this is not an empirical model and its use is not to provide optical closure but rather to identify and understand the biophysical drivers of phytoplankton optics and their contribution to an observable signal in the context of different water types. Here, the prominent model attributes are briefly underscored: The eukaryote model is explained first, then adjustments required for a vacuolate prokaryote version.

The EAP model is a fully physics-based two-layered spherical model that calculates biophysically linked phytoplankton absorption and scattering characteristics from a single particle. This calculation is undertaken from first principles i.e. from the imaginary part of the refractive index of a particle, reflecting the primary light-harvesting pigments of a variety of particle types (phytoplankton groups). The imaginary part of the refractive index approximately represents that portion of light that is absorbed by the cell, and the real part of the refractive index represents that portion of light which is scattered. The real part of the RI can also be related to cellular carbon content^16,17^. IOPs are calculated at high spectral resolution between 400 and 850 *nm* and are integrated over an entire size distribution of choice^18^, simulating the dominant optical characteristics of natural phytoplankton assemblages.

It is known that optical variability in phytoplankton is driven by particle size, pigment quantity and type, cellular material, shape and internal structure, fine-scale morphology, aggregation and physiological adaptations to the environment^19–21^. In the EAP model, particle size is a primary determinant of the optical properties. Because each set of EAP IOPs represents the optical properties integrated over an entire assemblage, the particles’ combined set of optical properties is designated as that of the distribution effective diameter^18^. This feature makes the model particularly useful for size-based Phytoplankton Functional Type (PFT) investigations, while being able to retain some second order accessory pigment and physiological variability. However, given the current interest in determining the extent of the capability of hyperspectral satellite radiometry to identify detailed spectral features of phytoplankton communities, the dataset presented here groups phytoplankton according to their dominant accessory pigment profiles, with some size variability as may be expected in natural assemblages. Pigment-driven variability is introduced via the choice of imaginary refractive index (representing unpackaged *in vivo* pigment absorption) and the *c*_*i*_ (representing intracellular Chl *a* density).

Variability in other biophysical attributes within a population can also be represented in the model, allowing investigation into further sources of optical variability. This capability includes the ability to change the shape and size range of the size distribution itself (e.g. a measured size distribution), adjust the ratio of core to shell sphere volumes, and change the *c*_*i*_ of the cells in the distribution, e.g. according to their size. It should be noted, however, that the model is not intended as a full representation of phytoplankton optical complexities, and there is certainly ecologically significant natural variability in phytoplankton IOPs that is not addressed by the model.

It should be borne in mind that significant variability in phytoplankton IOPs has been observed as resulting from physiological changes due to growth state^19^, responses to growth irradiance, nutrient availability and water temperature, and diel cycles^22–24^. While the model is able to reproduce some of this variability with the manipulation of the *c*_*i*_, some consideration should be given to the time and spatial scales on which these changes occur, and appropriate allowance made for the associated uncertainties. Additionally, a potentially large source of uncertainty is in the spherical shape and aggregation of particles, both of which can significantly impact upon a population’s IOPs^25–27^ although increasing shape complexity beyond the coated sphere model has been shown not to significantly reduce uncertainty^28^.

### Eukaryote model: (Phytoplankton Groups 1–15)

For eukaryotic particles, a core sphere represents the cytoplasm (which contains approximately 80% water, and is almost colourless), while an outer sphere represents the more refractive chloroplast, where the pigmented material (generally Chl *a* in the largest part) is also strongly absorbing.

#### Imaginary refractive index and its relationship with Chl a absorption and the package effect

The spectral nature of a phytoplankter’s imaginary refractive index is primarily represented by the measurement of absorption by its pigments in solution, i.e. unpackaged^29^. This spectrum serves as the primary input into the EAP model: it serves as the imaginary refractive index of the outer chloroplast sphere.

The magnitude of the imaginary refractive index is not important initially, just the shape; Chl *a*-specific absorption (*a*^∗^_*ϕ*_) is then constrained at 675 *nm* to reflect the theoretical maximum absorption by unpackaged Chl *a*^30^. Chl *a* is the dominant light harvesting pigment at 675 *nm* in most phytoplankton, and the resulting relationship between the theoretical maximum absorption and the magnitude of the imaginary refractive index takes into consideration the (inputted, variable) intracellular Chl *a* density (*c*_*i*_) of this pigment. There are some more recent estimates of this maximum theoretical absorption which exceed that of Johnsen *et al*.^31,32^, but increasing this value results in higher *a*^∗^_*ϕ*_, while we have found 0.027 *m*^2^ *mg*^*−1*^ to be appropriate for most phytoplankton that we have worked with.

This relationship is unique to the EAP model, and effectively results in the imaginary part of the refractive index being numerically linked to the specified *c*_*i*_ (see^22^ and others). This relationship is incorporated into the calculation of the magnitude of the imaginary refractive index of the chloroplast layer *n*^′^_*chlor*_ (outer sphere), based on the assumption that the cytoplasm layer (inner sphere) has no significant absorption at 675 *nm* (please note the typo in Applied Sciences paper^10^, where π is mistakenly in the numerator).

1 $${n}_{chlor}^{{\prime} }\left(675\right)=\frac{675}{{n}_{media}}\frac{{c}_{i}{a}_{sol}^{* }(675)}{\pi 4{V}_{v}}$$

where *n*_*media*_ = 1.334 and *V*_*v*_ is the relative chloroplast volume, *c*_*i*_ is the intracellular Chl *a* density, and *a*^∗^_*sol *_*(675)* is the *a*^∗^_*ϕ*_ at 675 *nm* of Chl *a* in solution, i.e. unpackaged^4^.

The effect of constraining the unpackaged absorption in this way is to establish a quantitative relationship between *c*_*i*_ and the cell volume; a relationship that is biophysically consistent as the cell size varies^4^. This approach results in an effectively decreasing *a*^∗^_*ϕ*_ with increasing size, observable in the resulting optics as the “package effect”^33,34^. *c*_*i*_ has been shown to vary not only with cell size but also with irradiance, as photophysiological responses of phytoplankton to light and nutrient limitation result in changes in cellular pigment density, which can be a major driver of the IOPs at small cell sizes and under dynamic light/nutrient regimes^35,36^.

#### Biophysically consistent derivation of real RI shape

A Hilbert transform filter allows a real signal to be transformed into its complex representation, and is used here to derive a real refractive index from the scaled imaginary refractive index. The full description of this derivation can be found in^4^.

It is the spectral shape of the real RI that is the desired product of the Hilbert transform, and the magnitude can be parameterised explicitly using available observations and/or models, also detailed in^4^. For most generalised phytoplankton groups, a real RI for the chloroplast of 1.10 is considered appropriate^4,16^.

The core imaginary RI represents absorption by the cytoplasm and approximates an exponential spectral shape of a weakly absorbing non-organic compound (it is primarily composed of water)^37^. Again, the Hilbert transform of the imaginary RI gives a corresponding real RI spectral shape, which is then scaled to 1.02^38^.

The model bases no calculations on an equivalent homogenous sphere, but for further understanding on how the RIs relate to one another, the Gladstone-Dale relations can be employed to give the refractive indices of an equivalent homogenous particle, based on the relative contribution of each layer of the sphere according to the volume it comprises:

The Gladstone Dale relations define the relationship between a combined particle refractive index and its density as the proportional contribution (by mass) of the component refractivities:

2 $$\frac{\left(n-1\right)}{p}={k}_{r}\;m$$

where *n* is the combined refractive index, *p* is the density, *k*_*r*_ is the component refractivity and *m* the proportional mass. With *m* = *Vp*, a volume equivalence can be implemented for the real and imaginary parts of the combined RI, where *V*_*c*_ and *V*_*s*_ represent the proportional contribution of the core and shell spheres to the total homogenous sphere volume, respectively:

3 $${k}_{hom}={k}_{core}* {V}_{c}+{k}_{shell}* {V}_{s}$$

4 $${n}_{hom}={n}_{core}* {V}_{c}+{n}_{shell}* {V}_{s}$$

giving the homogenous equivalent imaginary refractive index *k*_*hom*_, and the homogenous equivalent real refractive index *n*_*hom*_. This affords comparison with modelled and measured cellular RI data reported in the literature.

**Fig. 1:**
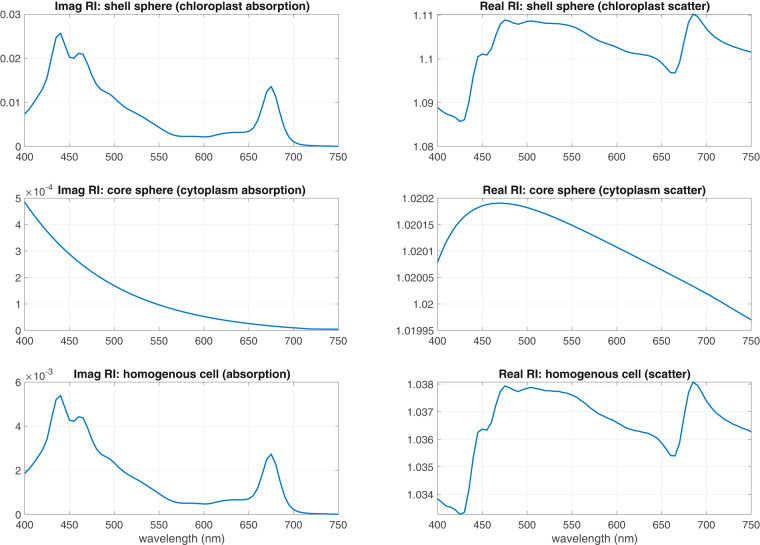
Complex Refractive Indices of core and shell spheres, and their homogenous sphere equivalents.

Note how the magnitude of the overall imaginary refractive index has dropped (Fig. 1), revealing that as only a small proportion of the whole cell, the overall absorption by pigments is significantly reduced relative to their measurement in solution. This is an important observation central to the model: it is the foundation of the package effect - enclosing the absorptive pigment inside a cell structure. The implementation of the package effect is thereby incorporated as a fundamental part of the model.

**Fig. 2:**
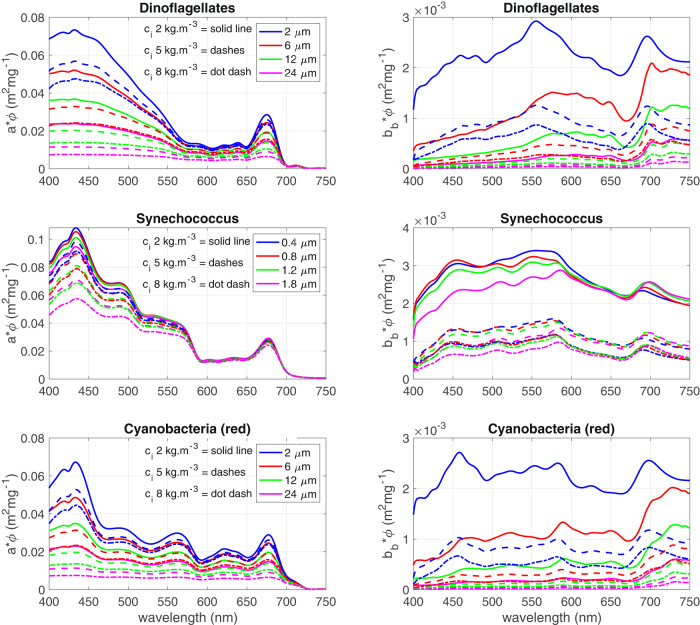
Chl *a*-specific absorption and backscatter for 3 eukaryotic phytoplankton groupings displaying distinct diagnostic pigments (fucoxanthin & peridinin, phycoerythrin and phycocyanin for Dinoflagellates, *Synechococcus* and Cyanobacteria respectively). These examples represent theoretical variability in assemblage effective diameter (*D*_*eff*_) and *c*_*i*_. The impact of *c*_*i*_ variability on the IOPs is particularly remarkable for small cells i.e. *Synechococcus*.

#### Complex refractive indices of core and shell spheres as input into D’Milay

The d’Milay code “Scattering by a stratified sphere” ^39^ is written in Fortran, and wrapped into Python for execution in the EAP model. The input into the d’Milay routines for the subsequent calculation of the bulk optical efficiency factors resulting from the contributions of each of the two layers of the sphere, are the complex RIs of the core and shell in turn:

5 $${m}_{shell}={n}_{shell}-{k}_{shell}* 1j$$

6 $${m}_{core}={n}_{core}-{k}_{core}1j$$

A full set of optical scattering and extinction efficiency factors are then calculated by the d’Milay routine, followed by the calculations of the backscatter probability for each particle, the IOPs, and then the integration of the IOPs over a specified size distribution. Fully spectrally and angularly resolved Volume Scattering Function and phase functions are included in the output.

Central to these calculations is the concept of Chl *a* specificity: a size distribution described only by shape (i.e. no cell counts) can be normalised to a total Chl *a* concentration of 1 *mg* *m*^*−*3^, by attributing a *c*_*i*_ to each cell. Likewise, a distribution specified by counts per unit volume as well as size bins can produce IOPs representing the total Chl *a* concentration of the assemblage.

Note also, that the effect on absorption with cell size is the further implementation of the package effect: as pigment density stays the same but the cell size increases, absorption becomes less efficient^29^. While the seminal Bricaud approach describes *a*^∗^_*ϕ*_ spectra appropriate for different biomass concentrations^40^, the EAP *a*^∗^_*ϕ*_ is driven by size differences^41^. As a semi-analytical model, the EAP can avoid the allometric assumption of increased biomass implying increased cell sizes. This capability is particularly important in the context of a changing ocean where previously described allometric relationships may no longer hold; for some applications it is critical that assumptions about biomass and cell size are avoided.

#### The carbon term

Generally IOPs are generated as Chl *a*-specific as per convention. However there is scope to parameterise the *C*_*i*_ too. This can be done within the Chl *a*-specific model in order to output total phytoplankton carbon for a distribution. But it can also be used to generate IOPs that are carbon-specific rather than Chl *a*-specific. It is widely acknowledged that Chl *a* concentration and biomass are not equivalent (e.g.^42–45^); carbon-specific IOPs may be useful towards improving productivity models^42^.

#### Linking chlorophyll to carbon

In the model, *c*_*i*_ and *C*_*i*_ can be parameterised independently from each other and independently from the RIs; however, there is some evidence to support a relationship with the imaginary and real RI respectively^16^, and these relationships are straightforward to implement according to the preference of the user.

Stramski (1999) presents such a relationship:

7 $${C}_{i}=3441.055\ast n(660)-3404.99$$

linking *C*_*i*_ to the real refractive index for two cultured phytoplankton species.

He also gives a relationship between *c*_*i*_ and the imaginary RI:

8 $${c}_{i}=996.86\ast n{\prime} \left(675\right)-1.17$$

(in Eqs. 3, 5, 6 of this paper we refer to the imaginary refractive index as *k*, this is the same as *n*′).

### Prokaryote Model (*Microcystis*-like)

**Fig. 3:**
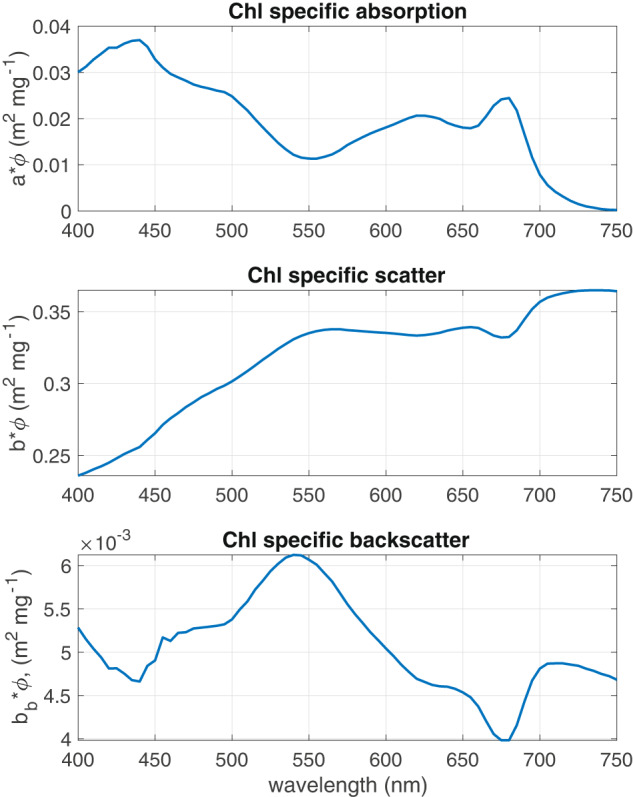
EAP Chl *a*-specific Inherent Optical Properties for *M. aeruginosa*, for a measured size distribution with a *D*_*eff*_ of 5.1 *um*.

The cellular arrangement of cyanobacteria and *M. aeruginosa* in particular provides a unique opportunity for the two layered model to investigate the influence of vacuole substructure. The standard assignment of the two spherical layers of the model to chloroplast and cytoplasm respectively, is less suitable to prokaryotic cyanobacteria. The thylakoids in cyanobacteria are not arranged in strict membrane-bound chloroplasts but rather occur in the intracytoplasmic membrane towards the periphery of the cell (the so-called chromatoplasm). Given this cellular arrangement, the opportunity arises for the core layer to be assigned to a vacuole-like particle, while assigning the shell layer to that containing the photosynthetic thylakoids and the cytoplasm^46^. This is based on the assumption that the vacuole can be adequately simulated as a single homogeneous particle of spherical shape; the full derivation of the refractive indices used for the prokaryote model is described^46^. The elevated scatter resulting from the addition of the vacuole is clearly noticeable in Fig. 3.

### Phytoplankton groups (PGs)

70 culture measurements of *in vivo* Chl *a* specific pigment absorption were collected from multiple laboratories^46–50^. The dataset includes 60 different species, with some overlap (10 measurements) between different labs (see Supplementary Material).

The dataset is arranged into Phytoplankton Groups based on spectral similarity in the measured species-specific Chl *a*-specific absorption (see^51^). These spectral similarities are driven by commonalities in the complement of accessory pigments present, resulting in spectral libraries loosely representing generalised taxonomic groups. It should be emphasised that these are not functional groups and are not intended to represent biogeochemical function. Within each group, a range of phytoplankton sizes are represented in the modelled dataset, so there is some potential for the implementation of a size-based functional approach, with an appropriately considered selection of IOPs. In this way, both pigment- and size-driven features in the IOPs may be investigated - within the scope of the dataset, in itself constrained by the available measurements.

The diatom species in the measured dataset are identifiable as either pennate or centric, and despite their spectral signature known to be dominated by light-harvesting pigments Chl *a* and fucoxanthin, there are sufficient spectral differences in absorption between the two types that they can be grouped separately. Pennate diatoms contain a raphe which may influence the optics, while centric diatoms present variations in accessory and photo-protective pigments such as Chl *c*, zeaxanthin, diadinoxanthin, diatoxanthin, and beta-carotene, resulting in ‘bumpier’ spectral absorption between 420 and 600 *nm*. In this dataset these groups are designated ***Diatoms (Pennate)*** and ***Diatoms (Centric)***. The dinoflagellate species are all joined together as a single PG ***Dinoflagellates*** due to spectral similarity. Other than Chl *a* and fucoxanthin, this group is uniquely dominated by the pigment peridinin. Species identifiable as ***Pelagophytes***, ***Raphidophytes*** and ***Eustigmatophytes*** have their own groups, all containing Chl *a*, Chl *c*, and fucoxanthin; however they differ in their contribution of accessory and photoprotective pigments. Two families of Haptophyte species are distinguished - ***Haptophytes: Pavlovaceae*** and ***Haptophytes: Prymnesiaceae***. These groups have similar pigment complements but the latter can be distinguished by the additional presence of 19’ butanoyloxyfucoxanthin and 19’ hexanoyloxyfucoxanthin. (Note that haptophytes outside of these families may display further variability - these are the only families represented in the measurements and thus available for the dataset). ***Chlorophytes*** represent a more ubiquitous class of green algae which can be found in marine, freshwater and brackish environments, whereas ***Prasinophytes*** represent green algae traditionally found more in the open ocean and that are usually of small size. They differ spectrally primarily due to variability in their intracellular concentration of pigment Chl *b*. ***Cryptophytes*** are represented as a single group that is characterized mainly by Chl *a* and Chl *c*, as well as a range of light harvesting pigments called phycobilins which give them their unique spectral signature. ***Rhodophytes***, or red algae, contain water-soluble pigments called phycobilins (phycocyanobilin, phycoerythrobilin, phycourobilin and phycobiliviolin), which are localized into phycobilisomes and give red algae their distinctive color. They also include Chl *a*, beta-carotene, and zeaxanthin. Cyanobacteria are separated into four groupings based on spectral characteristics. The two major subdivisions are a “blue-mode,” ***Cyanobacteria (blue)*** which are dominated by the pigment phycocyanin that uniquely absorbs strongly at 620 *nm*, and a “red-mode” ***Cyanobacteria (red)*** that are more dominated by phycoerythrin. ***Prochlorococcus*** and ***Synechococcus*** are also considered red-mode cyanophytes, but are identified here as individual groups due to their global ubiquity and relevance to the carbon cycle.

**Fig. 4:**
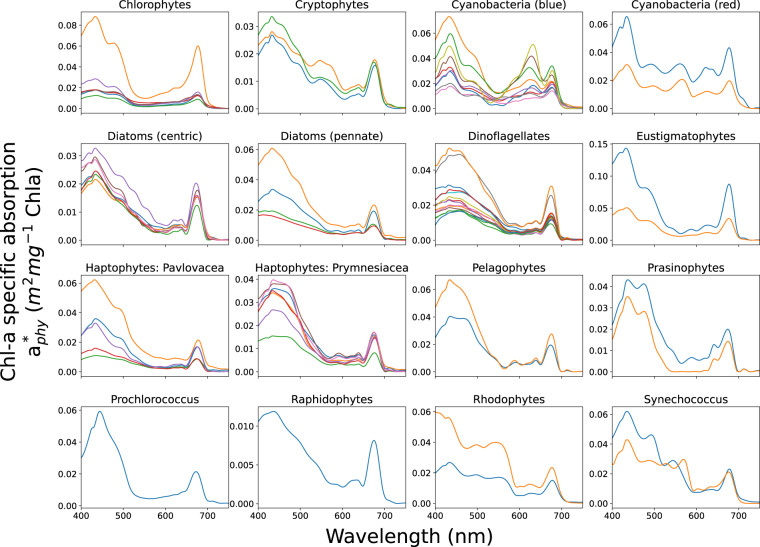
The 16 PG collections of the absorption measurements.

The final 16 Phytoplankton Groups derived from the absorption measurements are shown in Fig. 4, chosen primarily for spectral diversity (see Table 1). While spectral characteristics do not necessarily relate to biogeochemical function, it should be noted that cells of different sizes are represented within these pigment groupings allowing direct investigation into the robustness of pigment- and size-driven spectral characteristics respectively: in other words, understanding the characteristics of phytoplankton absorption and backscatter in terms of the combined effects of cell size and pigment composition on their contribution to the total optical signal of mixed phytoplankton assemblages.

**Table 1:** Output IOP dataset: 16 Eukaryote phytoplankton PGs, and 1 Prokaryote PG.

PG name	*D*_*eff*_ (*um*)	*c*_*i*_ (*kg m*^*−3*^)	PG description/comments
Diatoms (pennate)	6, 12, 24, 48	2, 5, 8	Pennate diatoms: high Chl *a* content, fucoxanthin, Chl *c*, beta carotene
Chlorophytes	2, 4, 6, 8	2, 5, 8	Green algae: marine, freshwater and brackish environments; beta carotene, Chl *b*, violaxanthin
Diatoms (centric)	6, 12, 24, 48	2, 5, 8	Centric Diatoms (fucoxanthin, beta carotene and photoprotective accessory pigments)
Cryptophytes	2, 6, 12, 24, 48	2, 5, 8	Cryptophytes - containing phycobilins, alloxanthin. Some can be as large as 50 um. Diagnostic pigment alloxanthin.
Cyanobacteria blue	2, 6, 12, 24	2, 5, 8	“Blue mode” cyanobacteria, dominant accessory pigment phycocyanin
Cyanobacteria red	2, 6, 12, 24	2, 5, 8	“Red mode” cyanobacteria, dominant accessory pigment phycoerythrin
Dinoflagellates	2, 6, 12, 24	2, 5, 8	Mixed dinoflagellates: Chl *a*, fucoxanthin, peridinin dominated
Eustigmatophytes	2, 6, 12, 24	2, 5, 8	Mostly freshwater, except Nonnochloropsis (2–4 um) Freshwater size range 2–25 um; beta carotene, violaxanthin
Haptophytes: *Pavlovaceae*	2, 6, 12, 24	2, 5, 8	Generalised Haptophytes: Chl c1, c2 and derivatives
Pelagophytes	1, 2, 3, 4	2, 5, 8	Small and widespread oceanic species, may form colonies, dominant accessory pigment fucoxanthin
Prasinophytes	2, 3, 4, 5,	2, 5, 8	Green algae: small oceanic types, Chl *b*, prasinoxanthin, violaxanthin, zeaxanthin
*Prochlorococcus spp*	0.4, 0.5, 0.7, 0.9	2, 5, 8	*Prochlorococcus* (small), no Chl *a; zeaxanthin, alpha carotene*
Haptophytes: *Prymnesiaceae*	1, 2, 3, 4	2, 5, 8	Small haptophytes, 19’ butanoyloxyfucoxanthin, 19’ hexanoyloxyfucoxanthin
Raphidophytes	12, 24, 48, 60	2, 5, 8	Large xanthophyte (yellow-green algae); Chl c1, c2, beta carotene, fucoxanthin, violaxanthin
Rhodophytes	2, 6, 12, 24, 48	2, 5, 8	Red algae – general. Wide size range; alpha carotene and derivatives, phycobilins
*Synechococcus spp*	0.4, 0.8, 1.2, 1.8	2, 5, 8	*Synechococcus* (small), phycoerythrin
*Microcystis*-like *spp (prokaryote)*	3, 4, 5, 6	2, 5, 8	*Microcystis spp*. (small, vacuolate), phycocyanin

Derivation of PG IOPs

**Fig. 5:**
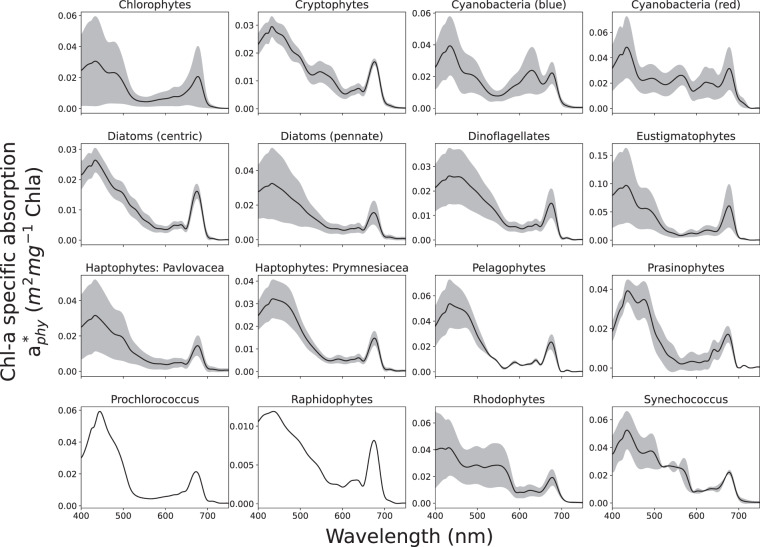
For each PG, a mean absorption spectrum was calculated from the absorption measurements, and this is used to represent the shape of the chloroplast imaginary refractive index input into the model to represent a generalised RI for each type (remembering that the magnitude is dictated by the parameterisation of pigment density). The greyed areas represent the range of variability in the original measurements, with the mean represented by the black line.

For each PG, a mean absorption spectrum was calculated from the absorption measurements (see Fig. 5), and this is used to represent the shape of the chloroplast imaginary refractive index input into the model to represent a generalised RI for each type (remembering that the magnitude is dictated by the parameterisation of pigment density). For the real RI, literature values were used as described previously for the eukaryote model. The greyed areas in Fig. 5 represent the range of variability in the original measurements, with the mean represented by the black line. Using a single mean imaginary RI (and a constant real RI) as input for each PG into the model means that variability in the resulting IOPs must be represented by cell size and by *c*_*i*_. The IOPs made available in this dataset are modelled for 4 or 5 different assemblage effective diameters per group, estimated to approximately represent natural cell size variability within each group. Each set of IOPs is integrated over a full size distribution with a standard normal shape and relatively narrow effective variance of 0.6, as would be appropriate for a monospecific culture.

It is critical to note that modelling choices regarding parameterisation of the inputs may result in IOPs that are not likely typically, or even possibly, observed in natural waters. *c*_*i*_ is a major driver of the IOPs but the documented range of variability in healthy cultures is quite small (0.5–6 *kg* *m*^*−*3^)^52^. However, the effects of photoacclimation on *c*_*i*_ are not well documented, and so the modelled range goes beyond that found in the literature^52^, to 8 *kg* *m*^*−*3^. So it needs to be clear that the IOPs provided are in order to represent a range which encompasses that of natural variability, and which is neither constrained by nor limited to the small selection of laboratory measurements available to parameterise the fundamental properties of the different groups.

## Data Records

The IOP dataset is available at Github: https://github.com/lisllain/EAP-model/tree/main/data/ and at Figshare^53^

A single folder is provided. The first .csv file (alphabetically) is labelled “ALL_INVIVO_MEANS.csv” and gives the mean derived imaginary refractive index for each PG. Imaginary RIs are given for 380 to 900 *nm* at 1 *nm* resolution. It should be noted however that values <385 *nm* and >800 *nm* are modelled using simple slope extrapolation, and are provided only to reduce edge effects on the Hilbert transforms.

A separate csv file is given for each PG, named as such:

Chlorophytes.csv

Cryptophytes.csv

Cyano_blue.csv

Cyano_red.csv

Diatoms_Centric.csv

Diatoms_Pennate.csv

Dinoflagellates.csv

Eustigmatophytes.csv

Haptophytes_Pavlovaceae.csv

Haptophytes_Prymnesiaceae.csv

Microcystis.csv

Pelagophytes.csv

Prasinophytes.csv

Prochlorococcus.csv

Raphidophytes.csv

Synechococcus.csv

Each csv has columns named for the IOP (“a”, “b”, or “bb”); the *c*_*i*_ in *kg m*^*−*3^ (2, 5 or 8); and the effective diameter of the assemblage: e.g. “a_Ci_2_Deff_6” gives chl-specific absorption for *c*_*i*_ = 2.0 *kg* *m*^*−*3^ for an assemblage with effective diameter of 6 micron. IOPs are given from 400 to 850 *nm* at 1 *nm* spectral resolution.

The PGs are represented by an appropriate range of assemblage effective diameters, and 3 different *c*_*i*_ parameterisations as listed in Table 1.

Full list of outputs available from the EAP model code as provided on Github:

Efficiency factors Qa, Qb, Qbb

Sigma a, Sigma b, Sigma bb

Absorption, Scatter, Backscatter for the total assemblage (Chl *a*-specific)

Backscatter probability function

Volume Scattering Function (1800 angles, all wavelengths)

Phase functions at each angle, each wavelength

The assemblage size distribution in counts per size bin (Chl *a*-specific)

The volume of cells in each size bin (Chl *a*-specific)

The total volume of cells in the distribution (Chl *a*-specific)

Total carbon in the assemblage (Chl *a*-specific)

## Technical Validation

There are unavoidable uncertainties in IOPs due to lack of empirical measurements of biophysical quantities (size distributions, intracellular pigment density, spheroid approximation of particle shape and so on). The impact of shape and aggregation on backscatter has been described^25^ and these results could be parameterised towards an estimate of uncertainty.

It is also well known that IOPs vary with an order of magnitude under physiological stress^34–36^. With all of this variability in mind, it should be remembered that the intention behind the model is to investigate causal relationships between pigment absorption profiles, particle size and density parameters and the resulting observable optical signal. The model is optimally employed when the investigative goals are parameterised with intention, e.g. size- vs pigment-driven variability in dinoflagellates, and when optical closure is not the object.

Due to the very reason this dataset is so useful, it is difficult to validate the scattering-driven phytoplankton IOPs directly. While there is confidence in phytoplankton absorption measurements and they match well with their modelled counterparts, backscatter measurements are notoriously difficult to perform and challenging to quality control. It should be emphasised that the most valuable capability of the EAP model *does not* lie in simulating the IOPs of individual species. Its power lies in the ability to represent broad scale changes/differences in phytoplankton IOPs as the main drivers of pigment, cell size and *c*_*i*_ are varied. It is not intended to be a species-specific model. That being said, the only IOP data possibly useful for model validation is from cultures, and therefore species-specific.

**Fig. 6:**
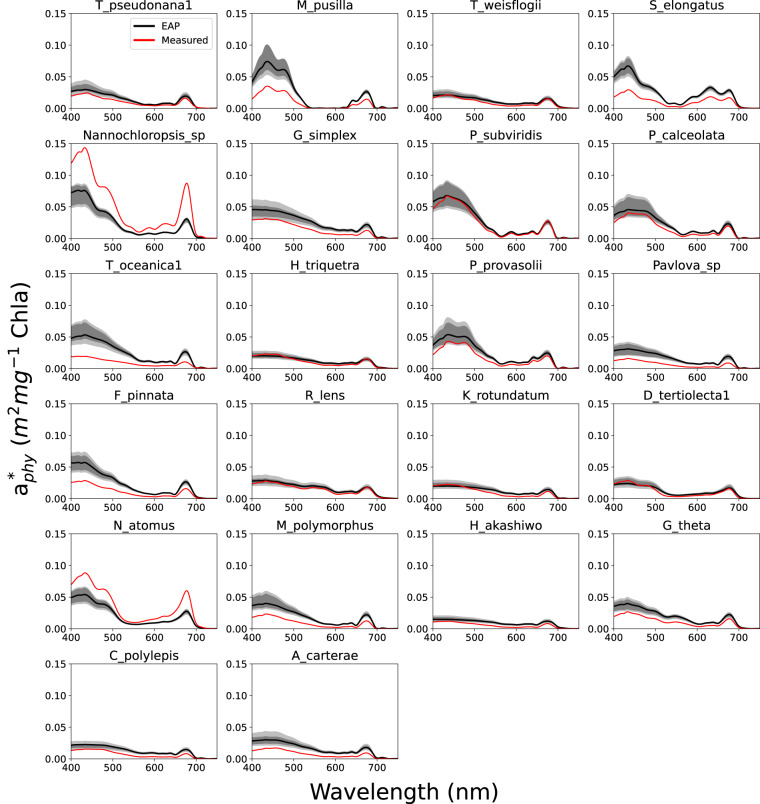
EAP modelled Chl *a*-specific absorption vs measured Chl *a*-specific absorption for individual species with corresponding ESD and *c*_*i*_ measurements available^48^. The grey ranges represent variability in the model driven by small variations in size and *c*_*i*_.

This species-specific IOP validation is presented in Fig. 6 (absorption), 7 (scatter) and 8 (backscatter). These species were chosen due to the availability of ESD and *c*_*i*_ measurements alongside their IOPs (see Supplementary Material). The species shown here depended entirely on the availability of the measured data, and so are not systematically representative of phytoplankton classes. They also represent homogenous assemblages of one species, an unusual if not impossible occurrence except in culture. Measured IOPs are drawn in red, while a range of corresponding EAP values is denoted in greyscale, with the median spectrum as a black line. The darker grey shaded area represents the ranges of the middle 50% of the spectral library, while the lighter grey represents the outer 50%. These spectral libraries were modelled using a small range of *c*_*i*_ and ESD around the laboratory measured values, to provide some level of uncertainty. These ranges are also shown in the Supplementary Material.

**Fig. 7:**
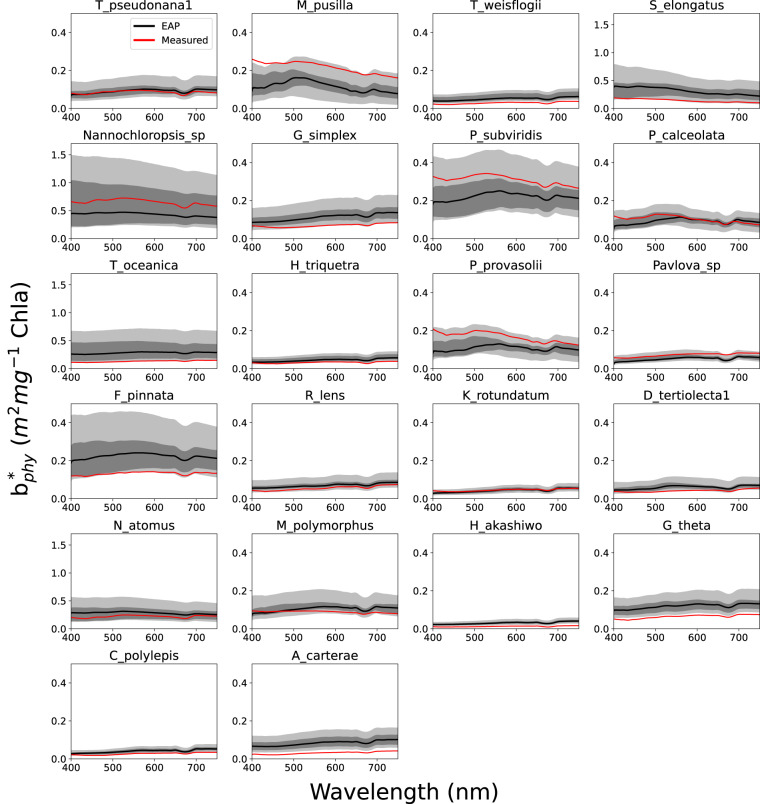
EAP modelled Chl *a*-specific scatter vs measured Chl *a*-specific scatter for individual species with corresponding ESD and *c*_*i*_ measurements available^48^. The grey ranges represent variability in the model driven by small variations in size and *c*_*i*_.

**Fig. 8:**
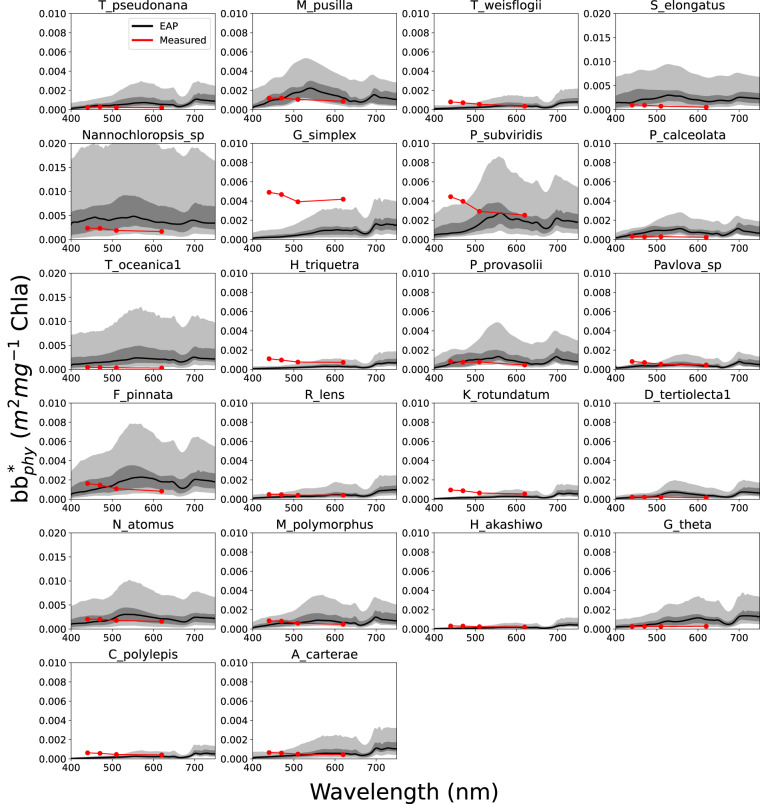
EAP modelled Chl *a*-specific backscatter vs measured Chl *a*-specific backscatter for individual species with corresponding ESD and* c*_*i*_ measurements available^48^. The grey ranges represent variability in the model driven by small variations in size and *c*_*i*_.

Generally, the capability of the model is well observed, with the magnitude of error of the 675 *nm* absorption peak fitting largely within a few micron uncertainty in the range of modelled cell sizes. There are varying possible explanations for larger mismatch in absorption in the blue, relative vs absolute error and so on. Given the unique shape and structure of the cells of individual species, uncertainty due to these features is exacerbated in the corresponding monospecific modelled IOPs - as is uncertainty in any of the laboratory measurements (cell size, *c*_*i*_). When modelling a more mixed assemblage comprising many different shapes and structures, these effects are less obvious. It is emphasised again that the aim of this dataset is *not* to reproduce species specific IOPs accurately, but to provide spectral libraries representing the dominant spectral features resulting from pigment, physiological state and size variability within and between the phytoplankton groupings.

It is noticeable in Fig. 8 that the shape of EAP and measured phytoplankton backscatter differ considerably in the blue, with elevated backscatter at short wavelengths evident in the measurements. It is understood that while phytoplankton themselves are known not to backscatter maximally in the blue, much smaller accompanying particles such as detritus or bacteria are more likely to be the origin of these features (D. Stramski, *pers comm*). These features may also originate from the sensor itself, with the measurement taken at a specific angle and then extrapolated to hemispherical backscatter using a chi factor^54^.

Further to these examples of individual species, a radiometric validation of the phytoplankton component of the EAP model is presented in Lain *et al*.^41^. With the phytoplankton simulations being the most challenging in terms of particle complexity, it can be concluded that a model demonstrated as successful in overwhelmingly phytoplankton-dominated waters addresses the phytoplankton component accurately.

Measured spectral phytoplankton absorption was compared against the retrieval of phytoplankton absorption by inversion of the EAP model (coupled with Hydrolight to produce a simulated reflectance dataset) and high levels of correlation were observed^5^, with low RMSE and standard error between the entirely independent absorption spectra. Validation of the backscatter once again was not possible due to lack of *in situ* measurements, but it was reasoned that due to the good agreement in absorption, the high level of convergence in the inversion algorithm, and the low level of optical contribution from non-algal sources in the extreme Case 1 Benguela region, there is likely a high level of validity in the phytoplankton backscatter estimates.

The model input parameters for *Microcystis spp*. were tested against various measurements found in the literature but ultimately the validity of the resulting prokaryote IOPs was determined by an optical closure exercise based on modelled *R*_*rs*_ and the observation that measured *R*_*rs*_ was overwhelmingly dominated by cyanobacteria, with small optical contributions from non-algal sources^6^. The methodology described elucidates on how the model can be tuned in an iterative fashion to achieve the most realistic balance of input parameters within documented ranges.

## Usage Notes

### This section is optional

#### Dataset strengths and limitations

It is not the intention of the authors to present this dataset as a solution for determining community structure from hyperspectral satellite data – aside from being extremely limited in terms of phytoplankton groups, previous work with the EAP model has shown the requirement for significant biomass (>2 *mg* *m*^*−3*^) in order for most pigment signals to be retrievable from current satellite radiometry with confidence^10^. A unique feature of this dataset and indeed the model is that there is no inherent allometric assumption about cell size increasing with biomass^41^ and so in terms of signal causality, the effects on the IOPs of the assemblage size distribution, biomass and pigment composition can be investigated separately and systematically.

This approach has many advantages: the detection of small spectral features due to unique pigment absorption profiles can be tested for robustness in mixed assemblages, informing on the limits of PG detection from bulk optical measurements; such a dataset can also be valuable towards examining the cost-benefit of hyperspectral vs multispectral optical water-leaving measurements.

Note that to better represent mixed, low biomass open ocean assemblages, a decaying exponential size distribution made up of a mixture of phytoplankton IOPs would likely be more appropriate.

In this way it can be understood that even within pigment groupings, the sources of optical variability are many, and signal ambiguity can be significant (see Fig. 2). Small variations in the choice of model input parameterisations (well within the range of published values) can result in significant IOP effects. Natural variability in intracellular chl (due to species, nutrient stress, photoacclimation etc.), morphology/composition effects, cell aggregation, assemblage size distributions, intracellular carbon and other biophysical parameters is in constant flux and impossible to replicate precisely. As previously mentioned, the dataset is designed to encompass reasonable ranges of natural optical variability rather than to exactly reproduce them.

The primary anticipated utility of such a dataset is as a starting point for applications focusing on phytoplankton optical signal composition and causal signal variability and ambiguity (intracellular Chl *a*, cell size, intracellular carbon variability), development of techniques and algorithms for signal detection and the physical limitations of signal-to-noise in measurements made by satellite sensors, determining phytoplankton signal retrieval uncertainties in the context of radiometric measurement uncertainty, cost vs benefit type analyses (multi vs hyperspectral data), and for input into radiative transfer models towards identifying potential for signal detection at Top of Atmosphere or improving atmospheric correction capabilities. There is enormous value in synthetic datasets for the development and training of machine learning and AI models as they can represent biophysically reasonable ranges of variability in phytoplankton optics, thereby informing on which deep learning techniques are most appropriate and effective for the intended application in terms of structure and sensitivity.

## Supplementary information

Supplementary Material

## Data Availability

A Jupyter notebook of the EAP model code is available on Github: https://github.com/lisllain/EAP-model. It is shared under a GNU General Public License. Appropriate acknowledgement should be made when the model is used in publications. Matlab code for writing IOP input files for the Hydrolight 4-component user-defined IOP model is also available, as are discretised EAP phase function^2^ files for Hydrolight. These will be added to the Github in due course but are available on request in the interim.
