# Supplementary material for: Simulated Inherent Optical Properties of Aquatic Particles using The Equivalent Algal Populations (EAP) model
Source: Sci Data. 2023 Jun 24;10:412. doi: 10.1038/s41597-023-02310-z (PMC10290707; doi:10.1038/s41597-023-02310-z)
Supplement: Supplementary file 1 — Supplementary Material [file 41597_2023_2310_MOESM1_ESM.pdf]

Supplementary Material

Simulated Inherent Optical Properties of Aquatic Particles using The Equivalent Algal Populations (EAP) model

Detailed below are the species used to create the spectral libraries for each Phytoplankton Group (PG). Dominant accessory pigments are identified for each PG[52], but these are by no means exhaustive. ESD and c_i are the equivalent spherical diameter and intracellular Chl *a* density which were measured in laboratory. ESD and c_i denoted with V represent the ranges used in the modeling of spectral libraries for the validation with Vaillancourt et al., 2004 data (Fig.s 6 - 8).

Citations are as follows (full references in main article):

i. Clementson & Wojtasiewicz, 2019
ii. Stramski et al., 2001
iii. Vaillancourt et al., 2004
iv. Mathews & Bernard, 2013
v. Wojtasiewicz & Ston-Egiert, 2016

Species	Phytoplankton Group	Accessory pigments	ESD (μm)	c_i ($kg.m^{-3}$)	ESD_V (μm)	c_i_V ($kg.m^{-3}$)	Cite
Thalassiosira oceanica 2	Diatoms (pennate)	chl-c, fucoxanthin	n/a	n/a	n/a	n/a	i
Ditylum brightwelli	Diatoms (pennate)	chl-c, fucoxanthin	n/a	n/a	n/a	n/a	i
Chaetoceros curvisetum	Diatoms (pennate)	chl-c, fucoxanthin	7.73	n/a	n/a	n/a	ii
Thalassiosira oceanica 1	Diatoms (pennate)	chl-c, fucoxanthin	5.7	1.8	3 - 7	1.5 - 3.5	iii
Dunaliella tertiolecta 3	Chlorophytes	beta carotene, chl-b, violaxanthin	n/a	n/a	n/a	n/a	i
Tetraselmis sp	Chlorophytes	beta carotene, chl-b, violaxanthin	n/a	n/a	n/a	n/a	i
Dunaliella tertiolecta 1	Chlorophytes	beta carotene, chl-b, violaxanthin	7.59	n/a	n/a	n/a	ii
Dunaliella bioculata	Chlorophytes	beta carotene, chl-b, violaxanthin	6.71	n/a	n/a	n/a	ii
Dunaliella tertiolecta 2	Chlorophytes	beta carotene, chl-b, violaxanthin	5.6	9.4	3 - 7	9 - 11	iii
Nannochloris atomus	Chlorophytes	beta carotene, chl-b, violaxanthin	3.1	2.6	2 - 5	2.5 - 4.5	iii

Thalassiosira pseudonana 1	Diatoms (centric)	chl-c1, chl-c2, fucoxanthin, diatoxanthin, diadinoxanthin	3.99		n/a	n/a	ii
Chaetoceros calcitrans	Diatoms (centric)	chl-c1, chl-c2, fucoxanthin, diatoxanthin, diadinoxanthin	6	n/a	n/a	n/a	iii
Fragillaria pinnata	Diatoms (centric)	chl-c1, chl-c2, fucoxanthin, diatoxanthin, diadinoxanthin	3.6	3.2	2 – 5	3 – 5	iii
Minutocellus polymorphus	Diatoms (centric)	chl-c1, chl-c2, fucoxanthin, diatoxanthin, diadinoxanthin	2.9	6.7	2 – 5	6.5 – 8.5	iii
Thalassiosira pseudonana 2	Diatoms (centric)	chl-c1, chl-c2, fucoxanthin, diatoxanthin, diadinoxanthin	4	6.4	3 – 7	6 – 8	iii
Thalassiosira rotula	Diatoms (centric)	chl-c1, chl-c2, fucoxanthin, diatoxanthin, diadinoxanthin	18	n/a	n/a	n/a	iii
Thalassiosira weissflogii	Diatoms (centric)	chl-c1, chl-c2, fucoxanthin, diatoxanthin, diadinoxanthin	12.8	3	11 - 15	3 - 5	iii
Chroomonas fragarioides	Cryptophytes	alloxanthin	5.57	n/a	n/a	n/a	ii
Guillardia theta	Cryptophytes	alloxanthin	5.4	4	4 - 7	4 - 6	iii
Rhodomonas lens	Cryptophytes	alloxanthin	7	5.2	5 - 9	5 - 7	iii
Cyanobacterium sp 1	Cyanobacteria (blue)	phycocyanin	n/a	n/a	n/a	n/a	i
Microcystis aeruginosa	Cyanobacteria (blue)	phycocyanin	5	n/a	n/a	n/a	iv
Alexandrium marina	Cyanobacteria (blue)	phycocyanin	1.43	n/a	n/a	n/a	ii
Synechococcus elongatus	Cyanobacteria (blue)	phycocyanin	2.7	2.2	1 - 4	2.5 - 4.5	iii
Aphanizomenon flos-aquae	Cyanobacteria (blue)	phycocyanin	n/a	n/a	n/a	n/a	v
Anabaena sp	Cyanobacteria (blue)	phycocyanin	n/a	n/a	n/a	n/a	v
Synechocystis salina	Cyanobacteria (blue)	phycocyanin	n/a	n/a	n/a	n/a	v

Nodularia spumigena	Cyanobacteria (blue)	phycocyanin	n/a	n/a	n/a	n/a	v
Nostoc sp	Cyanobacteria (blue)	phycocyanin	n/a	n/a	n/a	n/a	v
Cyanobacterium sp 2	Cyanobacteria (blue)	phycocyanin	n/a	n/a	n/a	n/a	v
Phormidium sp	Cyanobacteria (red)	phycoerythrin	n/a	n/a	n/a	n/a	v
Pseudanabaena galeata	Cyanobacteria (red)	phycoerythrin	n/a	n/a	n/a	n/a	v
Alexandrium margalefi	Dinoflagellates	fucoxanthin, peridinin	n/a	n/a	n/a	n/a	i
Alexandrium minutum	Dinoflagellates	fucoxanthin, peridinin	n/a	n/a	n/a	n/a	i
Alexandrium affine	Dinoflagellates	fucoxanthin, peridinin	n/a	n/a	n/a	n/a	i
Alexandrium catenella	Dinoflagellates	fucoxanthin, peridinin	n/a	n/a	n/a	n/a	i
Alexandrium tamarense 2	Dinoflagellates	fucoxanthin, peridinin	n/a	n/a	n/a	n/a	i
Woloszynskia	Dinoflagellates	fucoxanthin, peridinin	n/a	n/a	n/a	n/a	i
Prorocentrum micans 2	Dinoflagellates	fucoxanthin, peridinin	n/a	n/a	n/a	n/a	i
Heterocapsa niei	Dinoflagellates	fucoxanthin, peridinin	n/a	n/a	n/a	n/a	i
Prorocentrum micans 1	Dinoflagellates	fucoxanthin, peridinin	27.64	n/a	n/a	n/a	ii
Amphidinium carterae	Dinoflagellates	fucoxanthin, peridinin	9.1	3.3	7 - 12	3 - 5	iii
Alexandrium tamarense 1	Dinoflagellates	fucoxanthin, peridinin	31	n/a	n/a	n/a	iii
Gymnodinium simplex	Dinoflagellates	fucoxanthin, peridinin	7.6	3.1	5 - 9	3 - 5	iii
Heterocapsa triquetra	Dinoflagellates	fucoxanthin, peridinin	13.4	3.8	11 - 15	3.5 - 5.5	iii
Katodinium rotundatum	Dinoflagellates	fucoxanthin, peridinin	7.1	8.6	5 - 9	8.5 - 10.5	iii

Nannochloropsis oculata	Eustigmatophytes	violaxanthin, beta carotene	n/a	n/a	n/a	n/a	i
Nannochloropsis sp	Eustigmatophytes	violaxanthin, beta carotene	2.9	0.94	2 - 5	1 - 3	iii
Pavlova lutheri 2	Haptophytes: Pavlovaceae	chl-c1, chl-c2 and derivatives	n/a	n/a	n/a	n/a	i
Pavlova pinguis	Haptophytes: Pavlovaceae	chl-c1, chl-c2 and derivatives	3.97	n/a	n/a	n/a	ii
Pavlova Lutheri 1	Haptophytes: Pavlovaceae	chl-c1, chl-c2 and derivatives	4.26	n/a	n/a	n/a	ii
Pavlova sp	Haptophytes: Pavlovaceae	chl-c1, chl-c2 and derivatives	3.5	13.3	2 - 5	13 -15	iii
Pelagomonas calceolata	Pelagophytes	fucoxanthin	1.9	9.9	1 -4	9 - 11	iii
Pelagococcus subviridis	Pelagophytes	fucoxanthin	2.6	3.1	2 -5	3 - 5	iii
Micromonas pusilla	Prasinophytes	beta carotene, chl-b, prasinoxanthin, violaxanthin, zeaxanthin	1.4	6.6	0.5 - 2.5	6.5 - 8.5	iii
Pycnococcus provasolii	Prasinophytes	beta carotene, chl-b, prasinoxanthin, violaxanthin, zeaxanthin	2	7.1	1 - 4	7 - 9	iii
Prochlorococcus sp	Cyanobacteria (red)	zeaxanthin (no chl-a), alpha carotene	0.66 - 0.7	n/a	n/a	n/a	ii
Phaeocystis sp	Haptophytes: Prymnesiaceae	chl-c3, 19’ butanoyloxyfucoxanthin, 19’ hexanoyloxyfucoxanthin	n/a	n/a	n/a	n/a	i
Prymnesium parvum	Haptophytes: Prymnesiaceae	chl-c3, 19’ butanoyloxyfucoxanthin, 19’ hexanoyloxyfucoxanthin	6.41	n/a	n/a	n/a	ii
Isochrysis galbana 2	Haptophytes: Prymnesiaceae	chl-c3, 19’ butanoyloxyfucoxanthin, 19’ hexanoyloxyfucoxanthin	4.45	n/a	n/a	n/a	ii
Chrysochromulina polylepis	Haptophytes: Prymnesiaceae	chl-c3, 19’ butanoyloxyfucoxanthin, 19’ hexanoyloxyfucoxanthin (also chl-c2)	6.4	9.5	9 - 11	5 - 9	iii
Isochrysis galbana 1	Haptophytes: Prymnesiaceae	chl-c3, 19’ butanoyloxyfucoxanthin,	4	n/a	n/a	n/a	iii

		19’ hexanoyloxyfucoxanthin					
Heterosigma akashiwo	Raphidophytes	chl-c1, chl-c2, beta carotene, fucoxanthin, violaxanthin	10.9	7.8	8 - 13	7.5 - 9.5	iii
Porphyridium cruentum 2	Rhodophytes	alpha carotene and derivatives, phycobilins	n/a	n/a	n/a	n/a	i
Porphyridium cruentum 1	Rhodophytes	alpha carotene and derivatives, phycobilins	5.22	n/a	n/a	n/a	ii
Synechococcus sp 2	Synechococcus	phycoerythrin	0.7 - 1.14	n/a	n/a	n/a	ii
Synechococcus sp 1	Synechococcus	phycoerythrin	n/a	n/a	n/a	n/a	v
